# Climatic drivers of melioidosis in Laos and Cambodia: a 16-year case series analysis

**DOI:** 10.1016/S2542-5196(18)30172-4

**Published:** 2018-08

**Authors:** Philip L Bulterys, Michelle A Bulterys, Koukeo Phommasone, Manophab Luangraj, Mayfong Mayxay, Sabine Kloprogge, Thyl Miliya, Manivanh Vongsouvath, Paul N Newton, Rattanaphone Phetsouvanh, Christopher T French, Jeff F Miller, Paul Turner, David A B Dance

**Affiliations:** a UCLA-Caltech Medical Scientist Training Program, David Geffen School of Medicine, Los Angeles, CA, USA; b Molecular Biology Institute, Los Angeles, CA, USA; c Department of Microbiology, Immunology, and Molecular Genetics, Los Angeles, CA, USA; d California NanoSystems Institute UCLA, Los Angeles, CA, USA; e School of Public Health, University of Washington, Seattle, WA, USA; f Lao-Oxford-Mahosot Hospital-Wellcome Trust Research Unit, Vientiane, Laos; g Centre for Tropical Medicine and Global Health, Nuffield Department of Medicine, University of Oxford, Oxford, UK; h Faculty of Postgraduate Studies, University of Health Sciences, Ministry of Health, Vientiane, Laos; i Cambodia Oxford Medical Research Unit, Angkor Hospital for Children, Siem Reap, Cambodia; j Faculty of Infectious and Tropical Diseases, London School of Hygiene & Tropical Medicine, London, UK

## Abstract

**Background:**

*Burkholderia pseudomallei* is the cause of melioidosis, a serious and difficult to treat infection that is endemic throughout the tropics. Melioidosis incidence is highly seasonal. We aimed to identify the climatic drivers of infection and to shed light on modes of transmission and potential preventive strategies.

**Methods:**

We examined the records of patients diagnosed with melioidosis at the Microbiology Laboratory of Mahosot Hospital in Vientiane, Laos, between October, 1999, and August, 2015, and all patients with culture-confirmed melioidosis presenting to the Angkor Hospital for Children in Siem Reap, Cambodia, between February, 2009, and December, 2013. We also examined local temperature, humidity, precipitation, visibility, and wind data for the corresponding time periods. We estimated the *B pseudomallei* incubation period by examining profile likelihoods for hypothetical exposure-to-presentation delays.

**Findings:**

870 patients were diagnosed with melioidosis in Laos and 173 patients were diagnosed with melioidosis in Cambodia during the study periods. Melioidosis cases were significantly associated with humidity (p<0·0001), low visibility (p<0·0001), and maximum wind speeds (p<0·0001) in Laos, and humidity (p=0·010), rainy days (p=0·015), and maximum wind speed (p=0·0070) in Cambodia. Compared with adults, children were at significantly higher odds of infection during highly humid months (odds ratio 2·79, 95% CI 1·83–4·26). Lung and disseminated infections were more common during windy months. The maximum likelihood estimate of the incubation period was 1 week (95% CI 0–2).

**Interpretation:**

The results of this study demonstrate a significant seasonal burden of melioidosis among adults and children in Laos and Cambodia. Our findings highlight the risks of infection during highly humid and windy conditions, and suggest a need for increased awareness among at-risk individuals, such as children.

**Funding:**

Wellcome Trust.

## Introduction

*Burkholderia pseudomallei*, the cause of melioidosis, infects an estimated 165 000 people each year throughout the tropics, killing approximately 89 000.^1^ Infections are acquired from contaminated soil and surface water, the environmental reservoirs of *B pseudomallei*, via percutaneous inoculation, inhalation, or ingestion.^2^ Consequently, people who have regular contact with soil, such as rice farmers, are at increased risk of melioidosis. Additionally, people with diabetes, chronic lung or kidney disease, or alcoholism are at increased risk of infection. After infecting mammalian hosts, *B pseudomallei* is capable of replicating intracellularly and spreading from cell to cell by inducing membrane fusion, thereby evading host immune responses.^3^, ^4^ *B pseudomallei* is also intrinsically highly drug resistant and infected patients require prolonged treatment regimens.^5^ Even with appropriate antibiotic treatment, case fatality rates can approach 50%.^2^ Most cases occur during rainy and humid months in endemic areas. Studies of the seasonal correlates of disease in various settings have consistently found that rainfall and severe weather events predict increased incidence.^6^, ^7^, ^8^, ^9^, ^10^ Rainfall is hypothesised to affect exposure by increasing bacterial burdens in topsoil via rising water tables, and severe weather events are thought to promote the formation of contaminated aerosols that can be inhaled.^11^, ^12^, ^13^ Indeed, soil bacterial counts seem to peak during the rainy season, although this has not been a consistent finding across all studies.^14^, ^15^ Close examination of climatic variables in relation to melioidosis clinical data might provide insights into the ecology of *B pseudomallei*, the importance of various modes of transmission, and potential preventive strategies. Additionally, identification of seasonal drivers of infection provides a method of estimating the incubation period, which is often difficult to ascertain because of limited serological and exposure data.^16^

Our objective was to investigate the climatic factors governing the strong seasonality of melioidosis in two highly endemic lower-income countries, Laos and Cambodia. In Laos, melioidosis is a common cause of community-acquired septicaemia, particularly among the rice-farming communities of the Mekong flood plain.^17^, ^18^, ^19^ 80% of the Lao population live in rural areas and a high proportion are subsistence rice farmers, placing much of the population at risk of exposure to *B pseudomallei*. Additionally, approximately 5·6% of the general Lao population is reportedly diabetic.^20^ In recent years, more than 100 people have been diagnosed with melioidosis each year at Mahosot Hospital, but this is probably an underestimate of the total melioidosis burden.^21^ Turner and colleagues^22^ uncovered a substantial burden of melioidosis among Cambodian children. The authors reported an annual incidence of 28–35 cases per 100 000 children in Cambodia (also probably an underestimate), and a case fatality rate of 72% among bacteraemic patients. Laos and Cambodia share a similar tropical monsoon climate, with a rainy season typically lasting from May to October, followed by a cool dry season from November to April. Temperatures typically range from 22°C in December and January to 30°C in April and May, and humidity and precipitation both peak in July and August at around 82% humidity and 400 mm per month of precipitation. We aimed to identify climatic predictors of infection in Laos and Cambodia, to identify subpopulations at risk of infection because of climatic events, and to estimate the melioidosis incubation period.

**Research in context**

**Evidence before this study**

We searched PubMed, without language restrictions, for articles published up to May 2, 2018, using the search terms “melioidosis”, “*Burkholderia pseudomallei*”, “climate”, “weather”, “environment”, “environmental”, and “seasonality”. We identified several studies examining the seasonality of melioidosis, and the link between climatic variables and melioidosis in Malaysia, Singapore, Taiwan, Thailand, and Australia. These studies identified a highly seasonal pattern of *B pseudomallei* infection, with rainfall, humidity, and severe weather events predicting melioidosis cases. There were no studies examining environmental or climatic drivers of melioidosis in Laos or Cambodia, nor did any study specifically examine drivers of infection in children.

**Added value of this study**

We examined all melioidosis cases diagnosed at Mahosot Hospital, Vientiane, Laos, from 1999 to 2015, and all cases diagnosed at the Angkor Hospital for Children, Siem Reap, Cambodia, from 2009 to 2013, in relation to high-resolution spatial and temporal climatic data for the corresponding periods, to identify factors driving human exposure. Laos and Cambodia are resource-limited countries with a heavy melioidosis burden, for which little data about melioidosis exist. We identified humidity, rainfall, and wind speed as strong independent predictors of infection, with an estimated incubation period of 1 week (95% CI 0–2). We uncovered several new features of melioidosis epidemiology, which have potential implications for prevention. Humidity was associated with localised infection, whereas high wind speed seemed to drive pulmonary and disseminated infections. A substantial proportion of our patients were children, for whom the burden and epidemiology of melioidosis are poorly understood. We found that children were at an approximately three-times higher odds of infection than were adults during months of high (≥80%) humidity.

**Implications of all the available evidence**

Our study demonstrates a substantial seasonal burden of melioidosis among adults and children in Laos and Cambodia. Our results suggest that humidity and wind drive exposure to *B pseudomallei* in both countries, and that patients typically present very soon after environmental exposure. Our results also indicate that humidity and wind drive distinct clinical presentations, specifically localised infection (humidity) versus disseminated infection (wind). Children appear to be at especially high risk of infection during periods of high humidity, for unclear reasons. Our findings suggest a need for increased awareness among at-risk individuals and clinicians of the risks of infection, especially in children, during highly humid or windy months.

### Methods

#### Study design and patients

**Figure 1:**
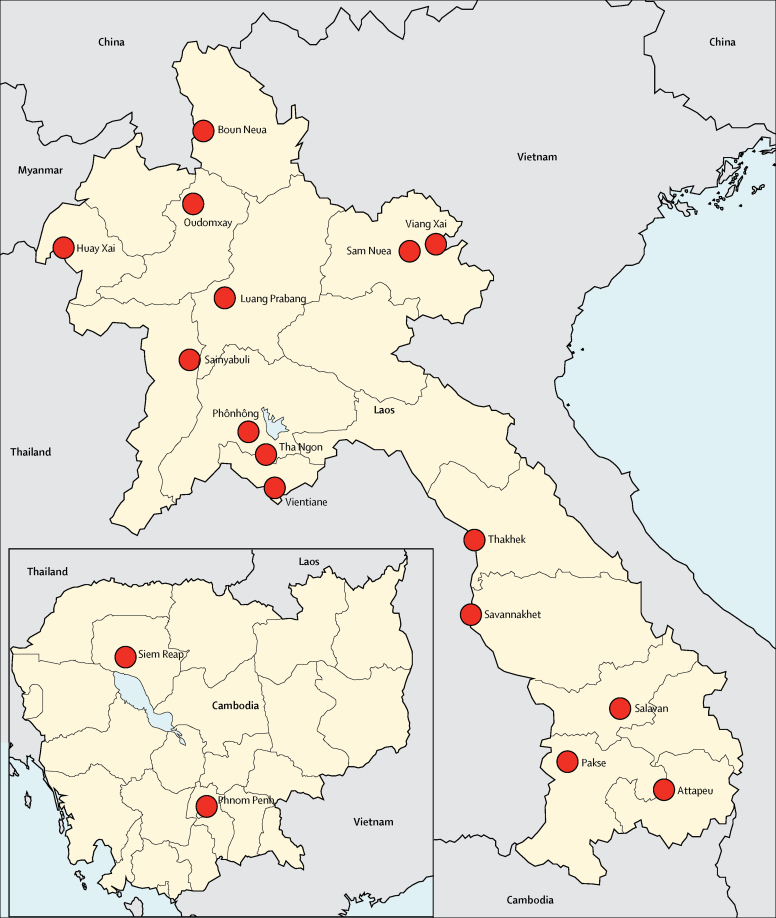
Locations of weather stations in Laos and Cambodia Climatic data were extracted from 15 weather stations in Laos and two stations in Cambodia (locations indicated by red circles). The majority of Lao patients (54%) lived in Vientiane or in the nearby areas of Phônhông (21%) and Tha Ngon (13%). The next most common areas of residence were Salavan (4%), Thakhek (2%), Pakse (1%), Attapeu (1%), Savannakhet (1%), and Sainyabuli (1%). 72% of Cambodian patients lived in Siem Reap province, and most lived closer to the Siem Reap weather station (88%) than to the Phnom Penh station (12%).

In this case series analysis, we examined all melioidosis cases diagnosed by the Microbiology Laboratory of Mahosot Hospital in Vientiane, Laos, from October, 1999, to August, 2015, and all culture-confirmed melioidosis cases presenting to the Angkor Hospital for Children in Siem Reap, Cambodia from February, 2009, to December, 2013. We identified patients with melioidosis using hospital microbiology records, and they were defined as patients in whom *B pseudomallei* had been isolated from at least one clinical specimen. We stratified patients by age (adult ≥18 years or child <18 years), sex, diabetic status (blood glucose at admission of ≥150 mg/dL *vs* <150 mg/dL, or medical history of diabetes), primary occupation (rice farmer or other), clinical presentation (localised or disseminated infection), and organ involvement (lung, liver, spleen, skin and soft tissue, or bone). Localised infection was defined as a single anatomical focus of infection, whereas disseminated infection was defined as two or more discrete anatomical areas of infection or *B pseudomallei* bacteraemia. We used the GPS coordinates of patients' home villages or communes to extract weather data from the nearest reliable weather station to the patient's home, using a global climate data repository that sources data from local meteorological stations and has been used in other epidemiological studies.^16^, ^23^, ^24^, ^25^ We extracted data from 15 weather stations in Laos and two weather stations in Cambodia (figure 1). If weather data from the nearest weather station were unavailable at the time of patient presentation, we extracted data from the next nearest weather station. We collected weather data for the 4 weeks leading up to a patient's presentation to the hospital, and included minimum, maximum, and mean temperature (°C), precipitation (mm), mean humidity (%), visibility (km), wind speed (km/h), and maximum sustained wind speed (km/h). Meteorological visibility refers to the transparency of air, and is subject to humidity and air-pollution levels.

This study was approved by the Oxford Tropical Ethics Committee, the Lao National Ethics Committee for Health Research, and the Angkor Hospital for Children Institutional Review Board.

#### Statistical analysis

We aggregated counts of patients with melioidosis by week and by month to allow sufficient resolution to generate a conditional estimate of the incubation period (week), and sufficient sample sizes to detect annual trends (month). We averaged (temperature, humidity, visibility, and wind speed) or summed (precipitation) weather variables over corresponding units of time. To identify factors associated with melioidosis cases, we performed univariable and multivariable negative binomial regressions by week and by month (to account for overdispersion of count data), with weather variables as the independent variables and the outcome being number of cases. We used weather data from the site with the most patients for regression analyses, using aggregated country-wide patient counts. For all other analyses, we used weather data from patient home villages. Variables that were significantly associated with melioidosis cases in univariable regression analyses were examined simultaneously in multivariable regression models. We performed a correlation analysis among significant variables to rule out multicollinearity. We also calculated the odds of melioidosis for different subpopulations, by stratification factor, during months of low, medium, and high humidity, and months of low, medium, and high wind speed to identify subpopulations that were especially susceptible to fluctuations in these variables. We selected cutoffs for humidity and maximum wind speed to obtain roughly equal case numbers in the low and high categories for each variable.

We fitted negative binomial regression models to examine hypothetical timing of *B pseudomallei* exposure. We estimated the date of exposure by subtracting a range of hypothetical incubation periods from the date of case presentation (exposure-to-presentation delays). By examining the likelihood scores corresponding with models fitted with different assumed incubation periods, we generated a conditional estimate of the melioidosis incubation period (conditional on the assumption that *B pseudomallei* exposure is directly linked to climatic variables, and that exposure led to disease).

We generated maximum likelihood estimates by calculating the negative log-likelihood (NLL) from the Akaike information criterion (AIC) of a given model, using the equation:

$$\Delta \text{NLL}=\frac{\Delta \text{AIC}}{2}$$

derived from the equation:

AIC=2κ-2log(L)

in which κ is the number of parameters (which was fixed across models) and ΔAIC is the AIC of a given model minus the lowest AIC value.^26^ We calculated a 95% CI using standard methods of likelihood profiling, and included all values that yielded log-likelihood scores within 1·92 units of the maximum score.^26^

We performed all statistical analyses using R (version 3.3.1). For regressions, we used the glm.nb function in the MASS package, with a log-link function. We performed a likelihood-ratio test to determine that the negative binomial regression model was required instead of a standard Poisson model due to overdispersion in the count data. We examined residuals and found that errors were not skewed across seasons.

#### Role of the funding source

The funder of the study had no role in study design, data collection, data analysis, data interpretation, or writing of the report. The corresponding author had full access to all the data in the study and had final responsibility for the decision to submit for publication.

### Results

There were 870 patients diagnosed with melioidosis in Laos, and 173 patients diagnosed in Cambodia during the study periods. Of the 870 patients in the Lao cohort, 357 (41%) of 869 were female, 330 (39%) of 840 had a medical history of diabetes, and 254 (45%) of 564 adults listed rice farming as their primary occupation. Median blood glucose on admission was 226 mg/dL (IQR 158–305) for patients with a history of diabetes and 98 mg/dL (79–149) for those with no history of diabetes. 480 (55%) of 866 patients presented with disseminated infection as opposed to localised infection (n=386, 45%), and the most commonly infected sites were the lung (n=309, 36%), skin and soft tissue (n=212, 25%), parotid gland (n=173, 20%), bone or joint (n=64, 7%), lymph node (n=61, 7%), spleen (n=59, 7%), liver (n=37, 4%), and urinary tract (n=15, 2%). The Cambodian cohort was comprised entirely of children, with a median age of 5·7 years (IQR 3·1–9·5), and 74 (43%) were girls. The majority (n=131, 76%) of Cambodian patients presented with localised infection as opposed to disseminated infection (n=42, 24%), especially of the skin and soft tissue (n=66, 38%), parotid gland (n=53, 31%), and lungs (n=33, 19%), and their clinical features have been previously reported in detail.^22^

**Figure 2:**
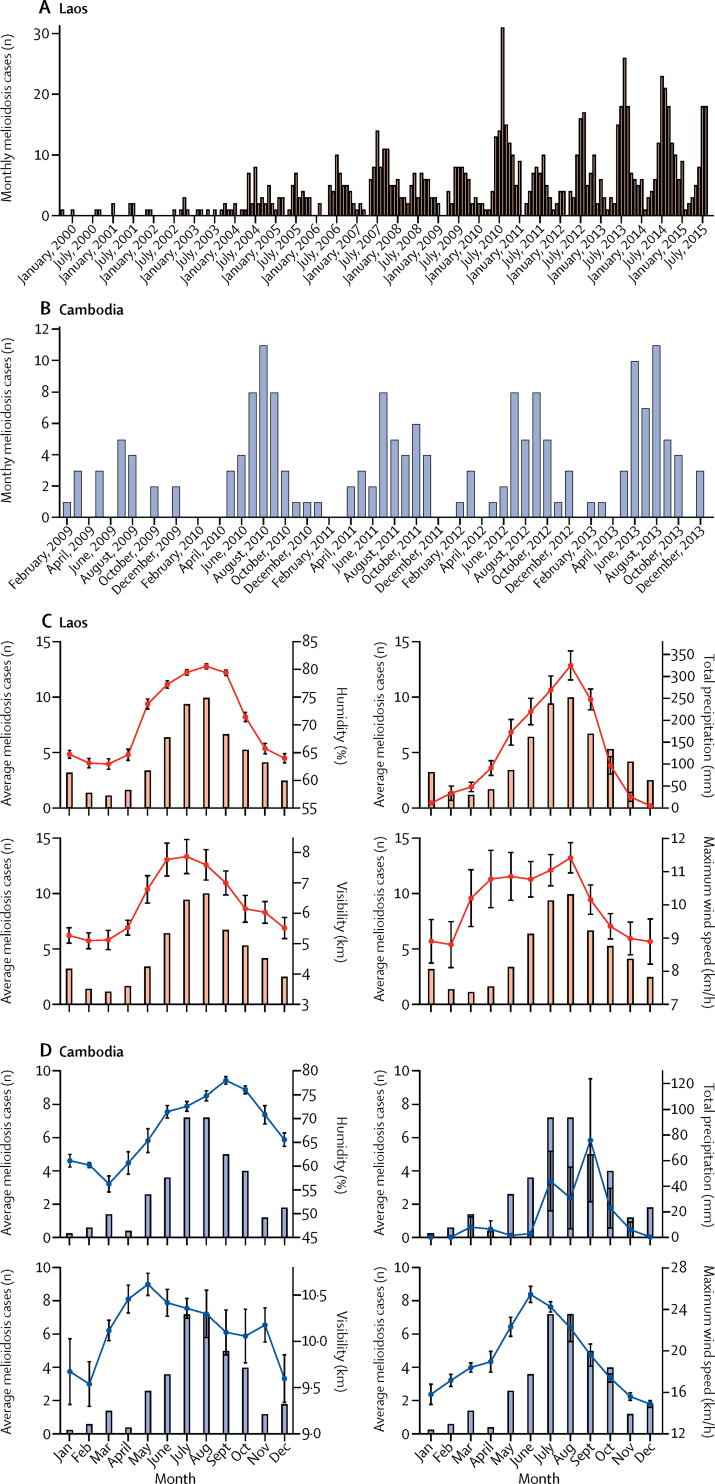
Monthly melioidosis cases in Laos and Cambodia, and association with climatic variables over time Monthly melioidosis cases in Laos, 1999–2015 (A) and monthly melioidosis cases in Cambodia, 2009–13 (B). Average monthly number of melioidosis cases and mean humidity, total precipitation, mean visibility, and mean maximum wind speed in Laos, 1999–2015 (C), and in Cambodia, 2009–13 (D). Error bars represent the SEM.

Weather data from the nearest weather station were unavailable for 6% of patients, for whom data were extracted from the next nearest. Vientiane in Laos and Siem Reap in Cambodia had the most patients, so these sites were used in the country-wide regression analyses. Melioidosis cases peaked annually during the rainy season (May–October) in both Laos and Cambodia, and declined during the dry season (November–April; figure 2). In the univariable regression analyses, melioidosis admissions in Laos were significantly associated with humidity (p<0·0001), precipitation (p<0·0001), low minimum temperature (p<0·0001), mean temperature (p=0·0084), low visibility (p=0·00043), maximum wind speed (p<0·0001), total days with rain (p<0·0001), and total days with thunderstorms (p<0·0001; table 1). When these variables were examined simultaneously in a multivariable regression model, only humidity (p<0·0001), low visibility (p<0·0001), and maximum wind speed (p<0·0001) remained significantly associated with melioidosis admissions (table 1). In univariable regression analyses, melioidosis admissions in Cambodia were significantly associated with humidity (p<0·0001), minimum temperature (p=0·0074), maximum wind speed (p=0·0013), total days with rain (p<0·0001), and total days with thunderstorms (p=0·00045). In a multivariable regression model examining humidity and total days with rain, both variables remained significantly associated with cases (p=0·010 for humidity and p=0·015 for total days with rain). When examined in a multivariable model with humidity, maximum wind speed also remained significantly associated with admissions (p=0·0070). When we ran multivariable models with the Lao cohort stratified by age, humidity (p<0·0001), low visibility (p<0·0001), and maximum wind speed (p=0·00020) were independent predictors of adult admissions, whereas humidity (p<0·0001), maximum wind speed (p=0·00025), and low minimum temperature (p=0·026) were independent predictors of paediatric admissions.

**Table 1:** Univariable and multivariable associations between monthly melioidosis cases and climatic variables

	**Univariable analysis**		**Multivariable analysis**	
	β coefficient (SE)	p value	β coefficient (SE)	p value
**Laos**				
Mean humidity, %	0·088 (0·01)	<0·0001	0·11 (0·0091)	<0·0001*
Precipitation, mm	0·0046 (0·00053)	<0·0001	−0·00033 (0·00084)	0·70†
Minimum temperature, °C	0·13 (0·030)	<0·0001	0·077 (0·19)	0·68*
Maximum temperature, °C	0·053 (0·042)	0·21	..	..
Mean temperature, °C	0·10 (0·039)	0·0084	−0·11 (0·20)	0·59*
Mean visibility, km	−0·17 (0·047)	0·00043	−0·36 (0·038)	<0·0001†
Mean wind speed, km/h	0·21 (0·13)	0·13	..	..
Maximum wind speed, km/h	0·24 (0·040)	<0·0001	0·14 (0·031)	<0·0001†
Total days with rain	0·069 (0·0086)	<0·0001	−0·026 (0·026)	0·31*
Total days with thunderstorm	0·091 (0·014)	<0·0001	0·016 (0·022)	0·45*
Total days with fog	−0·32 (0·20)	0·11	..	..
**Cambodia**				
Mean humidity, %	0·10 (0·017)	<0·0001	0·078 (0·030)	0·010‡
Precipitation, mm	0·0058 (0·0031)	0·062	..	..
Minimum temperature, °C	0·25 (0·094)	0·0074	0·16 (0·087)	0·068§
Maximum temperature, °C	−0·14 (0·081)	0·096	..	..
Mean temperature, °C	−0·089 (0·097)	0·36	..	..
Mean visibility, km	0·20 (0·27)	0·47	..	..
Mean wind speed, km/h	0·047 (0·10)	0·64	..	..
Maximum wind speed, km/h	0·12 (0·036)	0·0013	0·073 (0·027)	0·0070§
Total days with rain	0·090 (0·014)	<0·0001	0·051 (0·021)	0·015§
Total days with thunderstorm	0·099 (0·028)	0·00045	0·027 (0·026)	0·30§
Total days with fog	1·0 (1·0)	0·32	..	..

* Generated by simultaneous examination of all variables significantly associated with melioidosis cases in univariable analyses.

† Generated by simultaneous examination of variables significantly associated with melioidosis cases in multivariable analyses.

‡ Generated by simultaneous examination with total days with rain and maximum wind speed.

§ Generated by simultaneous examination with humidity with regard to melioidosis cases.

A correlation analysis of significant climate variables revealed a moderate positive correlation between weekly humidity and precipitation (*r*=0·66, *r*^2^=0·44), and weak or negligible correlations among other variables (table 2).

**Table 2:** Correlation coefficients (*r*) between climatic factors, Vientiane, Laos

	**Humidity**	**Precipitation**	**Visibility**	**Maximum wind speed**
Humidity	NA	0·66	0·35	0·03
Precipitation	..	NA	0·19	0·15
Visibility	..	..	NA	0·19
Maximum wind speed	..	..	..	NA

NA=not applicable.

The large sample size of the Lao cohort enabled comparison of patients by demographic and clinical features to determine which subpopulations were most strongly affected by seasonal variables. Specifically, we examined the odds of infection during months of low (<70%), medium (70–79%), and high (≥80%) monthly humidity, by each stratification factor (table 3). Children were almost three times more likely than were adults to become infected during months of high humidity (odds ratio [OR] 2·79, 95% CI 1·83–4·26), relative to low humidity months. The odds of infection during months of high humidity were not significantly different between women and men, or rice farmers and other professions (table 3). Patients with blood glucose concentrations of 150mg/dL or more at presentation were not at significantly greater odds of presenting with melioidosis during humid months than those with blood glucose levels lower than 150 mg/dL (table 3). Patients with a medical history of diabetes were less likely to present during high humidity months than patients without a medical history of diabetes (table 3). Localised infections were more likely to occur than were disseminated infections during months of high humidity (table 3). Lung and skin and soft tissue infections were also less common in months of high humidity (table 3). We examined these same subpopulations with respect to low (<10 km/h), medium (10–13 km/h), and high (≥13 km/h) maximum wind speeds, and identified a near-significant increased odds of lung infection in months of high wind speed (table 4). The odds of developing a disseminated infection were significantly higher than those of a localised infection during months of high wind speed (table 4).

**Table 3:** Odds of melioidosis for each stratification factor, by humidity, Laos

	**<70% humidity (low)**	**70–79% humidity (medium)**	**≥80% humidity (high)**
**Sex**			
Male	140	236	136
Female	89	166	102
Odds ratio (95% CI)	1 (ref)	1·11 (0·79–1·54)	1·18 (0·82–1·71)
**Age**			
Adult	186	295	144
Child	43	108	93
Odds ratio (95% CI)	1 (ref)	1·58 (1·06–2·36)	2·79 (1·83–4·26)
Primary occupation*			
Other	100	143	67
Rice farmer	74	116	64
Odds ratio (95% CI)	1 (ref)	1·10 (0·74–1·62)	1·29 (0·82–2·04)
**Blood glucose at admission**			
<150 mg/dL	89	130	66
≥150 mg/dL	86	146	74
Odds ratio (95% CI)	1 (ref)	1·16 (0·80–1·70)	1·16 (0·74–1·81)
**Medical history of diabetes**			
No	118	233	159
Yes	98	159	73
Odds ratio (95% CI)	1 (ref)	0·82 (0·59–1·15)	0·55 (0·38–0·81)
**Clinical presentation**			
Disseminated infection	128	246	106
Localised infection	98	157	131
Odds ratio (95% CI)	1 (ref)	0·83 (0·60–1·16)	1·61 (1·12–2·33)
**Organ involvement**			
Organs other than the lung	133	254	168
Lung infection	92	149	68
Odds ratio (95% CI)	1 (ref)	0·85 (0·61–1·18)	0·59 (0·41–0·86)
Organs other than skin and soft tissue	150	323	179
Skin and soft tissue infection	75	80	57
Odds ratio (95% CI)	1 (ref)	0·50 (0·34–0·72)	0·64 (0·42–0·96)

Data are n, unless otherwise indicated. Odds ratios are the odds of the lower variable (eg, female sex) over the upper variable (eg, male sex).

* Only adults included.

**Table 4:** Odds of melioidosis for each stratification factor, by wind speed, Laos

	**<10 km/h (low)**	**10–13 km/h (medium)**	**≥13 km/h (high)**
**Sex**			
Male	90	357	65
Female	80	235	42
Odds ratio (95% CI)	1 (ref)	0·74 (0·53–1·04)	0·73 (0·44–1·19)
**Age**			
Adult	131	414	80
Child	39	178	27
Odds ratio (95% CI)	1 (ref)	1·44 (0·97–2·15)	1·13 (0·64–1·99)
Primary occupation*			
Other	73	199	38
Rice farmer	48	172	34
Odds ratio (95% CI)	1 (ref)	1·31 (0·87–2·00)	1·36 (0·76–2·45)
**Blood glucose at admission**			
<150 mg/dL	59	188	38
≥150 mg/dL	60	204	42
Odds ratio (95% CI)	1 (ref)	1·07 (0·71–1·61)	1·09 (0·62–1·92)
**Medical history of diabetes**			
No	95	355	60
Yes	67	218	45
Odds ratio (95% CI)	1 (ref)	0·87 (0·61–1·24)	1·06 (0·65–1·75)
**Clinical presentation**			
Localised infection	90	256	40
Disseminated infection	80	333	67
Odds ratio (95% CI)	1 (ref)	1·46 (1·04–2·06)	1·88 (1·15–3·09)
**Organ involvement**			
Organs other than the lung	119	373	63
Lung infection	51	214	44
Odds ratio (95% CI)	1 (ref)	1·34 (0·93–1·94)	1·93 (0·98–2·70)
Organs other than skin and soft tissue	127	438	87
Skin and soft tissue infection	43	149	20
Odds ratio (95% CI)	1 (ref)	1·00 (0·68–1·49)	0·68 (0·37–1·23)

Data are n, unless otherwise indicated. Odds ratios are the odds of the lower variable (eg, female sex) over the upper variable (eg, male sex).

* Only adults included.

**Figure 3:**
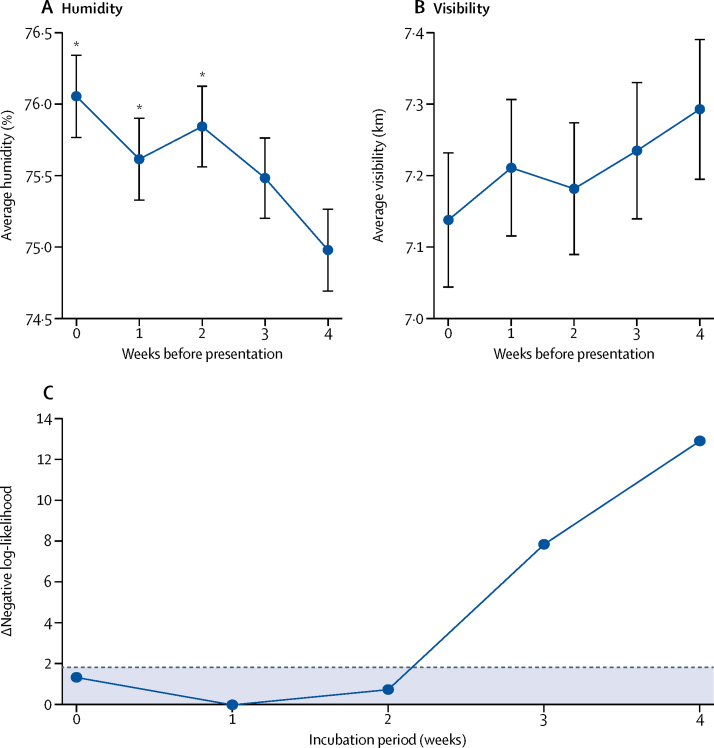
Mean weekly humidity (A) and visibility (B) during the 4 weeks before patient presentation, and maximum likelihood estimation of the incubation period (C), using patient data from Laos Error bars in (A) and (B) represent the SEM. (C) Negative log-likelihood values were obtained from negative binomial regression of weekly presentation data and mean humidity, incorporating exposure-to-presentation delays corresponding to incubation periods of 0–4 weeks. The maximum likelihood value (minimum negative log-likelihood) is 1 week (95% CI 0–2 weeks), indicating an incubation period of 2 weeks or less. The dashed line corresponds to a difference of 1·92 log-likelihood units from the optimum value. Points beneath this line fall within the 95% CI. *Significantly increased humidity compared with the fourth week before presentation.

Plots of the mean humidity and visibility 0–4 weeks before melioidosis presentation in Laos showed a clear pattern of increased humidity and decreased visibility in the 2 weeks before presentation (figure 3). We incorporated hypothetical exposure-to-presentation delays in our regression model to link melioidosis cases in Laos with weekly humidity, and compared the goodness of fit of these models for delays of 0–7 weeks to generate an estimate of the melioidosis incubation period that was conditional on the validity of the association with humidity. We selected humidity because it was the strongest predictor of cases in regression analyses. The likelihood scores for the regression model incorporating incubation periods of 0–4 weeks indicate a maximum likelihood estimate of the melioidosis incubation period of 1 week (95% CI 0–2; figure 3).

### Discussion

Elucidating the climatic factors that contribute to the marked seasonal incidence of melioidosis is crucial to understanding the epidemiology of this deadly disease, and to developing evidence-based preventive strategies for communities living in endemic areas. In this study, we examined climatic variables to identify drivers of infection in two highly endemic low-income countries, Laos and Cambodia. We uncovered several new features of melioidosis epidemiology, which have potential implications for prevention.

Several studies have examined the climatic predictors of melioidosis incidence,^6^, ^8^, ^9^, ^10^ but to our knowledge this is the first study to do so at high spatial and temporal resolution in Laos and Cambodia. Additionally, we believe this study is the first to examine the association between climatic variables and melioidosis for specific demographic and clinical subpopulations, including children. There is precedent for climatic factors driving the incidence of infectious disease, either by affecting host and vector dynamics or by directly affecting the pathogen. Notable examples of seasonal infections include influenza, malaria, bacterial meningitis, and penicilliosis.^16^, ^27^, ^28^, ^29^ Our findings are consistent with proposed mechanisms of *B pseudomallei* spillover from the environment into human populations (ie, inhalation of contaminated aerosols and contact with soil). We suspect that humidity, rain, and high-speed winds play an important part in exposing susceptible populations to *B pseudomallei*, partly by promoting the formation and spread of contaminated aerosols, or by facilitating growth of *B pseudomallei* at the soil surface. The strong association between melioidosis incidence and low visibility in Laos is consistent with this hypothesis, because high aerial water content would be expected to impair visibility. Our findings are consistent with other studies examining climatic drivers of melioidosis in Australia, Singapore, and Taiwan.^6^, ^8^, ^9^ In these settings, melioidosis case clusters were identified after periods of heavy rainfall and high humidity, and in Taiwan, wind speed also seemed to play a part, supporting the hypothesis that severe weather events drive exposure.

It is alarming that children are three-times more likely to become infected during months of high humidity, compared with adults, and this finding might reflect increased environmental exposure for children or immunological naivety, since *B pseudomallei* seroprevalence rates among children have been shown to increase with age.^30^ Ingestion of contaminated drinking water or swimming in contaminated bodies of water during these months could also account for this association.^31^ Alternatively, children could have a shorter incubation period and therefore present to the hospital sooner after exposure than adults. We were surprised to find that patients with a history of diabetes were less likely to present during months of high humidity than those without such a history, because diabetes is a known risk factor for melioidosis and one might suspect that these patients would be particularly susceptible during months with high environmental exposure.^32^, ^33^ One possible explanation would be that patients with known diabetes had better controlled disease than did those with potentially undiagnosed diabetes; however, significantly higher average blood glucose levels on admission among those with a history of diabetes seem to suggest otherwise. A more intriguing but perhaps less likely explanation for this paradoxical finding is that patients with a history of diabetes might have become infected with *B pseudomallei* in the past. For a subset of these patients, admission to the hospital could have represented reactivation of latent infection rather than primary infection, which would not be expected to follow a seasonal pattern. Another explanation could be that poor glycaemic control lowers the required inoculum to cause disease, or increases the risk of transformation of *B pseudomallei* infection to overt disease, enabling year-round exposures that lead to disease and therefore a non-seasonal pattern. Finally, although diabetes is not thought of as a seasonal illness, several reports from diverse climates have shown that haemoglobin A_1c_ (Hb_A1c_) concentrations increase during cold and dark months.^34^, ^35^, ^36^, ^37^ Although such a study has not been done in Laos or Cambodia, theoretical seasonal fluctuations in Hb_A1c_ (due to changes in diet, activity, or metabolism) could also help to explain our finding that patients with a history of diabetes were less likely to present than were patients without a history during the months of presumed highest exposure.

We found a non-significant association between lung infection and high wind speed, which is consistent with the hypothesis that wind contributes to inhalational exposure to *B pseudomallei*. We suspect that the OR for lung infection during high wind speed months did not reach significance because of the relatively low sample sizes. The finding that disseminated infection was more likely to occur during months of high wind speed could reflect higher rates of dissemination from the lung, as opposed to other foci. It was somewhat surprising that lung infections were less likely to occur than non-lung infections in very humid months. This result, in conjunction with the finding that localised infections were more likely than were disseminated infections during months of high humidity, might suggest that high humidity facilitates non-airborne exposure to *B pseudomallei*, perhaps by facilitating growth or survival in topsoil or air-exposed surfaces. Our estimate of the melioidosis incubation period is consistent with previous estimates,^6^, ^9^ and underscores the speed with which melioidosis can progress following environmental exposure.

Our study is limited in that it did not include data on all known risk factors for melioidosis, or exposure history. These data would have helped rule out potential confounders and provide an additional means of testing hypotheses about how patients become exposed to *B pseudomallei*. The rainy season is the time when people are most likely to be performing agricultural work, and this might have confounded some of our results. Although only 45% of adults in Laos reported rice farming as their primary occupation, the true proportion of study participants who farm rice was probably higher because many adults in Laos farm rice as a secondary occupation or hobby. This might explain our finding that rice farmers were not at increased risk of infection during humid or windy months. However, our finding that climate factors predicted case numbers at the weekly level suggests that these associations are real, since agricultural work would not be expected to vary by the week within the rainy season, or to increase during humid weeks. Another limitation is the fact that our study probably only included a fraction of the total population affected by melioidosis in Laos and Cambodia, because of limited access to health care. Additionally, our analysis did not account for potential fluctuations in population size over time and across regions, which could have affected the number of individuals at risk of infection at any given time.

This study benefited from a large sample size and detailed clinical and climatic data over a 16-year period in Laos and a 4-year period in Cambodia, enabling precise quantification of associations between melioidosis incidence and climatic variables. The study was also strengthened by inclusion of data from multiple study sites, and from multiple demographic groups, including children. Our findings should help guide prevention strategies in these endemic settings. In particular, during highly humid or windy weeks, it is reasonable to presume that the risk of exposure is significantly increased. Children seem to be at especially high risk on these occasions. Our results suggest that regular screening and treatment to control diabetes in adults and children, and avoidance of soil contact during humid and windy weeks, might reduce incidence. However, these approaches would be difficult to achieve in low-income settings such as Laos and Cambodia. Improved awareness of the risk of infection among at-risk communities and clinicians, especially during weeks of high humidity or wind speed, could reduce mortality due to melioidosis in these resource-limited settings. Further study will be needed to identify the specific human activities, and the specific ecological or environmental changes occurring in the soil, water, and air, that drive exposure to *B pseudomallei*, as well as the potential impact of climate change.
